# The Anti-Arthritic Activity of Diclofenac Lipid-Core Nanocapsules: Stereological Analysis Showing More Protection of Deep Joint Components

**DOI:** 10.3390/molecules28135219

**Published:** 2023-07-05

**Authors:** Nathalie Marte Ureña, Catiúscia Padilha de Oliveira, Silvia Stanisçuaski Guterres, Adriana Raffin Pohlmann, Oscar Tadeu Ferreira da Costa, Antonio Luiz Boechat

**Affiliations:** 1 Programa de Pós-Graduação e Imunologia Básica e Aplicada, Universidade Federal do Amazonas, Manaus 69077-000, Brazil; 2 Programa de Pós-Graduação em Ciências Farmacêuticas, Faculdade de Farmácia, Universidade Federal do Rio Grande do Sul, Porto Alegre 90610-000, Brazil; 3 Laboratório de Morfologia Quantitativa, Departamento de Morfologia, Instituto de Ciências Biológicas, Universidade Federal do Amazonas, Manaus 69077-000, Brazil; 4 Laboratório de Terapias Inovadoras, Departamento de Parasitologia, Instituto de Ciências Biológicas, Universidade Federal do Amazonas, Manaus 69077-000, Brazil

**Keywords:** diclofenac, nanoformulation, lipid-core nanocapsules, stereology, adjuvant arthritis

## Abstract

Diclofenac is the most prescribed nonsteroidal anti-inflammatory drug worldwide and is used to relieve pain and inflammation in inflammatory arthritis. Diclofenac is associated with serious adverse effects, even in regular-dose regimens. Drug delivery systems can overcome this issue by reducing adverse effects and optimizing their efficacy. This study evaluated the activity of lipid-core nanocapsules loaded with diclofenac (DIC-LNCs) in an experimental model of adjuvant-induced arthritis. The diclofenac nanoformulation was obtained via self-assembly. A stereological analysis approach was applied for the morphological quantification of the volume, density, and cellular profile count of the metatarsophalangeal joints of rats. Proinflammatory cytokines and biochemical profiles were also obtained. Our results showed that the diclofenac nanocapsule DIC-LNCs were able to reduce arthritis compared with the control group and the DIC group. DIC-LNCs efficiently reduced proinflammatory cytokines, C-reactive protein, and xanthine oxidase levels. Additionally, DIC-LNCs reduced the loss of synoviocytes and chondrocytes compared with the DIC (*p* < 0.05) and control groups (*p* < 0.05). These data suggest that DIC-LNCs have anti-arthritic activity and preserve joint components, making them promising for clinical use.

## 1. Introduction

Nonsteroidal anti-inflammatory drugs (NSAIDs) are the most commonly prescribed medications to treat pain and stiffness caused by rheumatoid arthritis [1]. NSAIDs act via the inhibitory cyclooxygenase (COX) enzymes and block the production of prostanoids (prostaglandins (PGs), prostacyclin (PGI2), and thromboxane (TX)), which promote pain, inflammation, and swelling of inflamed tissue [2].

Diclofenac is the most commonly prescribed NSAID and inhibits the isoforms of cyclooxygenase, COX-1, and COX-2 [3]. Diclofenac temporarily relieves RA symptoms and is usually used with disease-modifying antirheumatic drugs (DMARDs) and glucocorticoids to relieve pain and inflammation. Nonsteroidal anti-inflammatory drugs do not alter the progression of RA, but their long-term use has serious side effects [4]. Some adverse effects of NSAIDs include esophagitis, peptic ulcers, gastrointestinal bleeding, chronic kidney disease, and death, especially cardiovascular events. These side effects are caused by poor selectivity for inflamed tissues [3,5].

To overcome this difficulty, nanotechnology can be used to synthesize nanopharmaceuticals that can deliver drugs to targeted areas of inflammation, avoiding the possible side effects that drugs can cause in the system [6]. Studies have demonstrated the enhanced anti-inflammatory activity and strong modulation of proinflammatory mediators when used in nanostructured vehicles [7,8].

Lipid-core polymeric nanocapsules (LNC) are polymeric nanoparticles composed of a polymeric wall and organogel core containing a dispersion of sorbitan monostearate in medium-chain triglycerides stabilized by polysorbate 80 micelles [9]. Recent studies have demonstrated that diclofenac (acid form) loaded in lipid-core nanocapsules with high encapsulation efficiency is distributed mainly in the polymer watt of the LNC, which can decrease the dose required to elicit the necessary therapeutic activity and side effects. The use of LNC has shown many advantages, such as decreased TNF-α, IL-1, and IL-6 levels and increased IL-10 levels in an arthritis model using LNC-loaded indomethacin [10]. 

To understand experimental arthritis, many studies have used stereology to quantify the morphological changes in these models [11]. Stereology is “The body of methods for the investigation of three-dimensional space when only two-dimensional sections through solid bodies or their projections on a surface are available” [12]. The application of stereology allows for calculating volume, density, and counting of structures of one determinate object through analyzing two-dimensional images. Obtain accurate results with minimum bias by evaluating serial sections of the object of interest [13]. Studies have demonstrated the efficacy of this technique in analyzing experimental models of arthritis [14,15]. While conventional histology evaluates the qualitative hallmarks of joint inflammation, stereology can provide accurate quantitative information to understand the effects of treatment on key joint tissue components in experimental arthritis. We hypothesized that the diclofenac lipid-core nanocapsule aqueous formulation (DIC-LNC) preserves chondrocytes and synovial space and diminishes synovitis in arthritic joints. This study evaluated the anti-arthritic activity of DIC-LNCs in an experimental model of arthritis using stereological analysis. 

## 2. Results

### 2.1. Preparation of DIC-LNC Dispersed in Water

Lipid-core nanocapsules loaded with diclofenac, DIC-LNCs, and blank lipid-core nanocapsules (LNC) were white-bluish opalescent liquids with a Tyndall effect (visual aspect). The granulometric profile of the DIC-LNC and LNC obtained by laser diffraction (LD) analysis showed unimodal distributions with D[4,3] below 204 ± 46 nm (span < 1.7) for both formulations. No micrometer particle population was formed, regardless of whether the formulation contained diclofenac. Table 1 summarizes all the values obtained using the different techniques for DIC-LNC and LNC. These formulations were also analyzed by dynamic light scattering (DLS) to improve accuracy in the nanoscale range measurements. They showed a hydrodynamic mean diameter (Dh) below 170 nm with narrow polydispersity indices (PDI < 0.1). In addition to DLS, the hydrodynamic diameters of the formulations were obtained by nanoparticle tracking analysis (NTA). NTA determines the diameters of individual particles by tracking and sizing them and recording positional changes due to Brownian movement. The formulations showed Dh between 182 and 196, D90 below 309 nm, and particle number density between 4.76 × 10^12^ and 4.98 × 10^12^ nanocapsules mL^−1^ (Table 1).

After assessing the size distribution, the drug loading and encapsulation efficiency of the DIC-LNC were evaluated, showing drug content of 1.09 ± 0.10 mg mL^−1^ and encapsulation efficiency of 100%. The pH and zeta potential were determined, and no significant differences were observed between DIC-LNCs and LNC.

### 2.2. DIC-LNC Reduces Edema Formation at the Hind Paws

**Figure 1:**
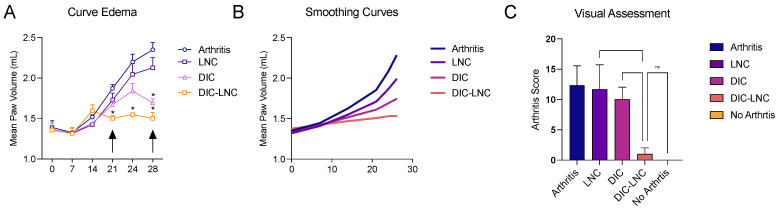
Arthritis evaluation and scores for twenty-eight days. (**A**) Arthritis kinetics, the black arrows indicate the maximum effects observed. (**B**) Edema curve with smoothing techniques. (**C**) Arthritis score. For convenience, at the figures the number of asterisks over the bars are the number of digits with significance after the decimal point (* significance at one digit after decimal point).

Figure 1 shows the edema formation (Figure 1A,B) and the treatment effects on hind-paw volume and arthritis scores (Figure 1C). DIC-LNC reduces paw volume compared to the control (*p* < 0.0001) and DIC (*p* = 0.05) at 28 days, mean reduction of −0.848 mL (95% CI −1.80 to −0.51). Moreover, the treatment effect of DIC-LNC appears to be early arthritis reduction on the 21st day compared to DIC (mean reduction −0.338 mL 95%CI −0.54 to −0.19, *p* < 0.0001).

### 2.3. DIC-LNC Reduces the Serum Levels of Proinflammatory Cytokines and CRP

**Figure 2:**
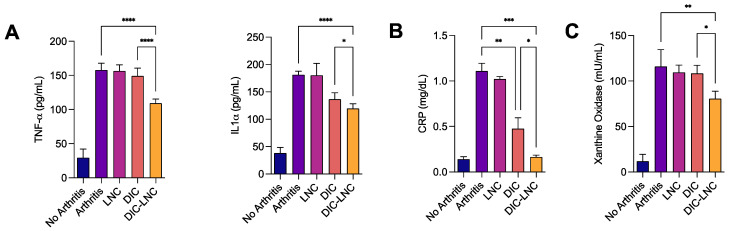
Serum inflammatory markers on day twenty-eight of the experiment. (**A**) Quantification of the proinflammatory cytokines TNF-α and IL-1α. (**B**) Serum levels of CRP for the DIC and DIC-LNC groups. (**C**) xanthine-oxidase as an oxidative stress marker. For convenience, at the figures the number of asterisks over the bars are the number of digits with significance after the decimal point (* significance at one digit after decimal point; ** significance at two digits after decimal point; *** significance at three digits after decimal point; **** significance at four digits after decimal point).

The serum levels of TNF-α were significantly lower in the DIC-LNC group compared with the DIC group using Sidak’s test (*p* < 0.0001; Figure 2A). The serum IL1α levels were similar for both groups when the comparison was performed using a Tukey’s test (*p* = 0.778); however, the linear test for trend indicated a significant trend (*p* = 0.0001). CRP and xanthine-oxidase levels were also lower in the DIC-LNC group than in DIC (Mann–Whitney U test, *p* = 0.0286, Figure 2B,C).

### 2.4. Absence of Liver and Renal Toxicity with DIC-LNC

**Figure 3:**
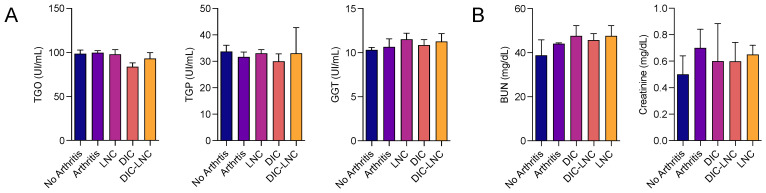
Liver and kidney toxicity biochemical markers. (**A**) Liver biochemical markers (TGO, TGP, GGT) of all five groups (Ul/mL). (**B**) Kidney biochemical markers (BUN, CRE) for all five groups (mg/dL).

Liver enzymes and renal function markers were evaluated to assess liver and kidney toxicity (Figure 3A,B). The use of DIC-LNCs did not alter the serum levels of these enzymes (*p* > 0.05) at the doses used in this study, suggesting no liver toxicity (Figure 3A). Following the same protocol, serum creatinine and blood urea nitrogen (BUN) serum levels were measured to evaluate renal toxicity (Figure 3B). It was also observed that DIC-LNCs did not alter the serum levels of BUN or creatinine compared with the other treatments (*p* > 0.05). 

### 2.5. Cavalieri’s Volume of Metatarsophalangeal (MTP) Joints

**Figure 4:**
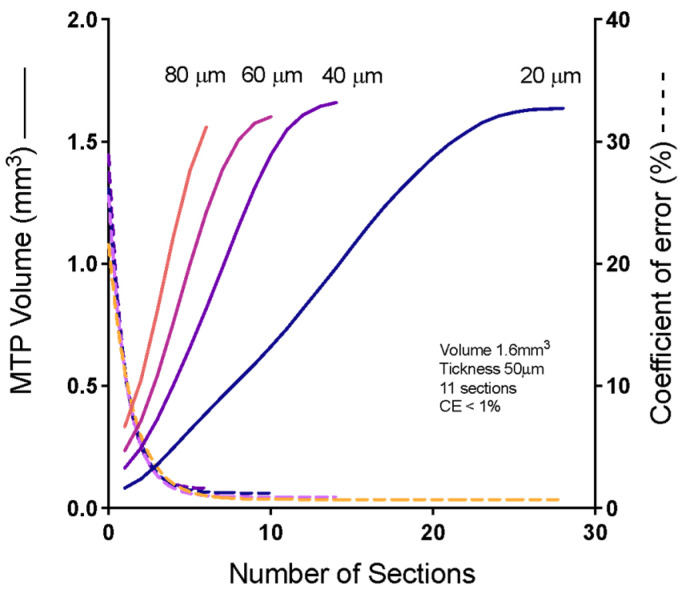
Relationship between Cavalieri’s volume and the coefficient of error. Demonstration of the relation between the number of serial sections (*x*-axis), the volume of Cavalieri (right y-axis), and the coefficient of error (left *y*-axis). Continuous lines indicate the volume, and dotted lines represent the error coefficient. Blue indicates 28 equidistant 20 mm serial sections; purple indicates 14 equidistant 40 mm serial sections; pink indicates 10 equidistant 60 mm serial sections; orange line indicates 16 equidistant 80 mm serial sections.

Figure 4 shows the effect of the number of sections on the efficiency and accuracy of determining the Cavalieri volume of the MTP joint. According to Cavalieri, a rat paw finger was histologically processed into resin and thoroughly sectioned to produce 28 sections evaluated for volume determination in a pilot study. A reductive process was applied to show the relationship between the smallest number of sections and a low coefficient of error (CE). Our results indicate that using 10 sections is as accurate as using 28 sections and is much more efficient.

**Figure 5:**
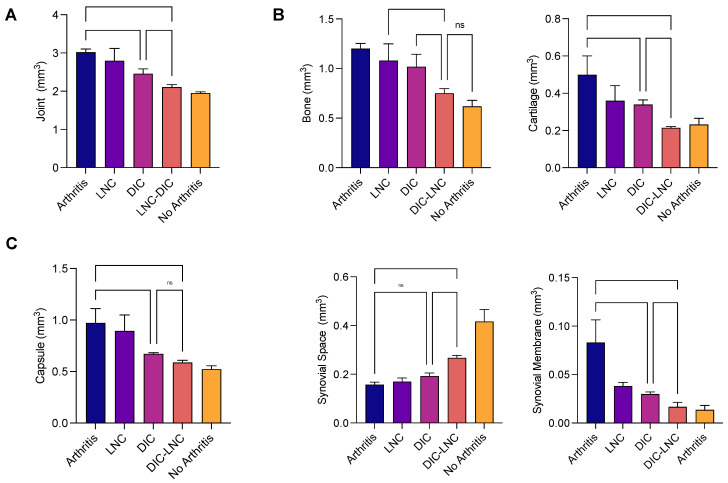
The volume of the MTP joint and each component. (**A**) MTP joint volume of all treated groups. (**B**) Absolute volume of the components of MTP of each treated group. (**C**) Volume of Capsule, Synovial Space, and Synovial Membrane. ANOVA significance *p* < 0.05.

Table 2 and Figure 5A present the results for MTP joint volume. The volume was higher in the arthritis group than in the diclofenac (*p* = 0.0007) and DIC-LNC groups (*p* < 0.0001). Most importantly, the DIC-LNC presented differences with the diclofenac group (*p* = 0.023), indicating that the DIC-LNC formulation was more efficient in controlling edema formation. These results demonstrate that DIC-LNCs effectively reduced joint volume, with values similar to those of the No Arthritis group.

### 2.6. Density of the Joint Components and Absolute Volume

Table 2 and Figure 5B summarize the results obtained by calculating the relative volume of each MTP component. The Arthritis group presented an elevated volume of cartilage (arthritis vs. DIC *p* = 0.002; arthritis vs. DIC-LNC *p* < 0.0001), bone (arthritis vs. DIC *p* = 0.142; arthritis vs. DIC-LNC *p* = 0.0002), capsule (arthritis vs. DIC *p* = 0.142; arthritis vs. DIC-LNC *p* < 0.0002), and synovial membrane (arthritis vs. DIC *p* = 0.0003; arthritis vs. DIC-LNC *p* = 0.0001) when compared with the diclofenac and DIC-LNC groups, respectively. Interestingly, cartilage volume expansion reflects both damages to the cartilage content by inflammation and marginal bone and cartilage formation.

Treatment with DIC-LNCs preserved the synovial space (*p* = 0.007) and diminished the volume of cartilage (*p* = 0.031), bone (*p* = 0.016), and synovial membrane (*p* = 0.0009, Brown-Forsythe ANOVA test due to unequal standard deviation and Dunnett’s T3 for multiple comparisons) when compared with the diclofenac group (Figure 5C). Together, these data demonstrate that DIC-LNCs caused a reduction in joint inflammation preserving joint elements. 

### 2.7. Surface Area of MTP Joints

**Figure 6:**
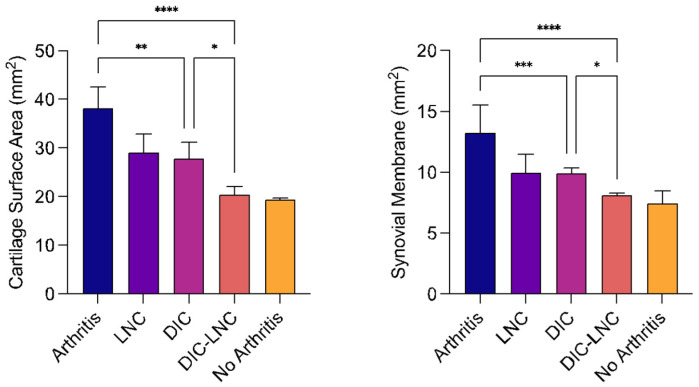
The surface area of MTP. The left panel presents the surface area of the cartilage. The right panel presents the surface area of the synovial membrane. ANOVA significance *p* < 0.05. For convenience, at the figures the number of asterisks over the bars are the number of digits with significance after the decimal point (* significance at one digit after decimal point; ** significance at two digits after decimal point; *** significance at three digits after decimal point; **** significance at four digits after decimal point).

Table 3 and Figure 6 show the results for the surface areas of the cartilage and synovial membrane of the MTP. Erosion at the cartilage surface is usually reflected in higher surface area measurements. However, a higher surface area was observed due to the inflamed synovial villosities. Thus, the Arthritis group presented the highest surface areas of cartilage (arthritis vs. DIC *p* = 0.001; arthritis vs. DIC-LNC *p* = 0.0001) and synovial membrane (arthritis vs. DIC *p* = 0.038; arthritis vs. DIC-LNC *p* < 0.0001) when compared with the diclofenac and DIC-LNC groups, respectively. As shown in Figure 6, treatment with DIC-LNCs restored the values of cartilage (*p* = 0.015) and synovial membrane (*p* = 0.009) compared with the diclofenac group. 

### 2.8. Quantification of Chondrocytes

**Figure 7:**
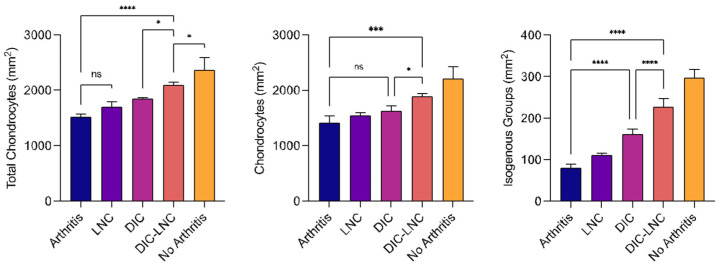
Cellular profile count of MTP chondrocytes. The left panel shows the total number of chondrocytes count. The middle panel shows the number of isolated chondrocytes. The right panel shows the number of isogenous groups of chondrocytes present after mitosis. ANOVA significance *p* < 0.05. For convenience, at the figures the number of asterisks over the bars are the number of digits with significance after the decimal point (* significance at one digit after decimal point; *** significance at three digits after decimal point; **** significance at four digits after decimal point).

Table 3 and Figure 7 summarize the results of the chondrocyte count. The No Arthritis group had the highest number of total chondrocytes. In contrast, the Arthritis group presented the lowest number of chondrocytes (*p* = 0.084, *p* = 0.002) compared to the diclofenac and DIC-LNC groups. The Arthritis group also presented a reduction in the number of isogenous groups (*p* < 0.001; *p* < 0.001) and the total number of chondrocytes (*p* = 0.004; *p* <0.001) compared with the diclofenac and DIC-LNC groups. The DIC-LNC group presented a higher number of isogenous groups (*p* < 0.001) and total chondrocytes (*p* = 0.004) than the DIC-LNC group.

## 3. Discussion

In this study, we aimed to evaluate the anti-arthritic activity of DIC-LNCs in an experimental model of arthritis using stereological analysis. To this end, a lipid-core nanocapsule containing diclofenac was formulated via interfacial deposition using a polymer method. In this study, we used quantitative morphological methods to demonstrate that diclofenac-loaded lipid-core nanocapsules (DIC-LNCs) can reduce paw inflammation and cytokine production, preventing synovitis, synovial space, and cartilage loss. Together, these results strongly support the anti-arthritic effects of DIC-LNCs.

For both formulations, DIC-LNCs and LNC, the polydispersity index values were lower than 0.1, indicating narrow size distributions of the nanocapsules, as shown by unimodal size distribution profiles. Moreover, DIC-LNC presented a mean diameter of 204 ± 46 nm, pH of 5.39 ± 0.16, Dh of 196 nm, and D90 of 309 nm obtained by NTA. These physicochemical parameters indicated that the nanocapsules were suitable for intravenous circulation. Lipid-core nanocapsules (LNC) are effective formulations for entrapped pharmaceuticals [16] and for delivering drugs to specific areas of inflammation, such as multiform glioblastoma [10], neuroinflammation [17], and chronic inflammation [18]. Furthermore, LNCs are nontoxic in experimental animal models [19]. Thus, these nanomaterials can efficiently deliver pharmaceuticals to specific areas while minimizing the side effects of drugs. 

Despite the success of DMARDS for rheumatoid arthritis treatment using a treat-to-target approach, pain and inflammation remain challenges in clinical practice [20]. Proinflammatory cytokines such as tumor necrosis factor-alpha (TNF-α), interleukin-1-beta (IL-1β), interleukin-6 (IL-6), and interleukin-17 (IL-17) can increase peripheral nociceptive neurons sensitization [21]. These cytokines are locally produced by proliferating synovial cells, such as macrophages and fibroblasts, and activated T lymphocytes in joint-inflamed synovia [22]. When inflammatory signals proliferate, synovial membrane cells infiltrate joint components and cause cartilage destruction, bone erosion, and joint space narrowing [23]. Eicosanoid production in inflamed synovia is related to pain sensitization and joint inflammation [4] and is stimulated by inflammatory cytokines [24]. 

It has been well known that proinflammatory cytokines especially enhance the expression of cyclooxygenase-2 (COX-2) and metalloproteinases (MMP) in human synovial fibroblasts (RASF) [25]. Inhibition of cyclooxygenase and leukotrienes reduces cartilage and bone erosion in both adjuvant arthritis and collagen-induced arthritis [26,27]. Moreover, recent investigations have shown that patients with RA with optimized treatment highly express cyclooxygenase in the synovial membrane [28]. When the human cyclooxygenase-2 gene is silenced on cultured synovial cells of rheumatoid arthritis patients, there is a reduction of prostaglandin E2 (PGE2), vascular endothelial growth factor (VEGF), IL-1β, and TNF-α [29]. Together, these data may account for the anti-arthritic effects of DIC-LNCs over conventional diclofenac in experimental arthritis in our study.

Stereological analysis has proven an augmentation of bone formation in arthritic experimental models. A group of researchers analyzed the bone surfaces of the joints of arthritic mice. They observed that the mineralized surfaces of bones were higher in arthritic mice (*p* < 0.001) than in the control group [11]. This may be explained by the production of TNF-α that promotes the differentiation of osteoclasts in the arthritic joints, which leads to the invasion of inflammatory tissue, imbalance of bone resorption/formation, and later remodeling of the bone [30].

Our study observed a higher bone volume in the arthritic joints than in the other groups. Additionally, the experimental model of arthritis used in this study, Adjuvant-Induced Arthritis (AIA), is an aggressive representation of RA that leads to ankylosis and joint malformations. AIA is characterized by soft tissue inflammation, marked bone loss, and new periosteal bone formation [31]. These data indicate that arthritic joints are involved in joint remodeling. 

A marked manifestation of rheumatoid arthritis is the synovial membrane [32]. In the synovial lining, there is an increase in the activity of fibroblast-like synoviocytes, which produce prostaglandins (PGs) and metalloproteinases (MMPs) that contribute to joint [33]. Synovitis has been related to the production of TNF-α, IL-17, IL-6, and IL-1. This proinflammatory cytokine group causes synovia hyperplasia and angiogenesis by activating macrophages, dendritic cells, and T and B lymphocytes [34]. 

Synovial membrane augmentation in RA patients has been studied using stereological analyses. Researchers have observed a significant increase in synovia compared to the control group [35]. In addition, by applying stereological analysis, Kristensen et al. studied synovial proliferation in arthritic rabbits. They observed that the arthritic joints of rabbits presented the highest scores for synovial proliferation and thickness compared to the control group [36]. Our results also showed an increase in the synovial membrane volume in arthritic joints compared to the other groups. These results are consistent with those reported previously. 

Drug nanoencapsulation may increase drug–tissue interactions and concentration or modify drug biodistribution [37]. For instance, ketoprofen-loaded polymeric nanocapsules can cross the blood–brain barrier by passive transport after in vivo treatment of glioblastoma in rats [38]. When nanocapsules are administered orally, their adhesion to the mucosa improves the performance of the drug nanosystem. Thus, the indomethacin ethyl ester lipid-core nanocapsules interact with the gastrointestinal tract to act as a mucoadhesive drug reservoir [39]. Nanocapsules are internalized together with the encapsulated substances into the endosomal compartment or micronuclei. Subsequently, the drug is released via simple diffusion, enzymatic polymer degradation, or redox-responsive polymer degradation.

Recently, a network meta-analysis showed that diclofenac was more likely to control pain and improve physical function, with adverse effects similar to those of other NSAIDs [40]. The therapeutic effects of diclofenac on arthritis are largely related to its synovial fluid concentration [41]. The synovial bioavailability of diclofenac, in its turn, may be limited by oral absorption and first-pass hepatic metabolism [41,42]. A high plasma concentration of diclofenac is a key pharmacokinetic element in the synovia. After six hours, with a single intravenous 75 mg injection, the diclofenac synovial concentration corresponded to 166% of plasma levels [41]. However, after the first-pass metabolism, approximately only 50% of the drug reaches the systemic circulation in an unchanged form, and this extensive metabolism accounts for diclofenac having poor oral bioavailability [43].

Moreover, after oral diclofenac intake, large interindividual differences in plasma concentrations have been observed [44]. These variations may account for therapeutic inefficiency due to low serum concentrations and the observed adverse reactions when higher serum levels were achieved. As previously reported, diclofenac nanocapsule formulations achieve higher serum concentrations than sodium diclofenac oral solutions with lower drug-induced organ damage [45]. 

## 4. Materials and Methods

### 4.1. Materials

Poly(ε-caprolactone) (PCL) (Mw = 65 000 g mol^−1^) and sorbitan monostearate (Span 60^®^) were supplied by Sigma-Aldrich Co. (Burlington, VT, USA). Caprylic/capric triglyceride (CCT) was obtained from Delaware (Brazil), and polysorbate 80 from Brasquim (Brazil). Sodium diclofenac was purchased from Galena (Brazil). All other chemicals and solvents were of analytical or pharmaceutical grade. All the reagents were used as received.

### 4.2. Preparation of the Lipid-Core Nanocapsules Formulations

First, neutral diclofenac (acidic form) was obtained using a previously described methodology [46,47], in which an aqueous medium containing sodium diclofenac was acidified with 5 mol L^−1^ of hydrochloric acid until turbidity was observed. The mixture was kept static to allow precipitation in a cooling bath, and the precipitate obtained was filtered and recrystallized using water: ethanol (1:1, *v/v*). The colorless crystals were characterized by infrared spectroscopy (FT-IR 8300, Shimadzu) with a resolution of 4 cm^−1^ and 64 scans, and the range of the frequency from 4000 to 500, presenting bands at wavelength numbers (cm^−1^) of 3300 (NH), 3200–2500 (OH), and 1710 (C=O).

The formulations containing or not containing diclofenac, denominated DIC-LNC and LNC, respectively, were prepared by interfacial deposition of a preformed polymer methodology as previously reported [9,47]. The organic phases, at 40 °C, composed of acetone (27 mL), PCL (0.100 g), sorbitan monostearate (0.038 g), capric/caprylic triglyceride (0.160 g), and diclofenac (0.010 g), were poured into the aqueous phase (53 mL) containing polysorbate 80 (0.077 g) at 40 °C. A translucent solution was obtained instantaneously, after which acetone was removed, and the solution was concentrated under reduced pressure at 40 °C. The final volume was adjusted to 10 mL by using a volumetric flask.

### 4.3. Physicochemical Characterization of the Formulations

#### 4.3.1. pH Measurements

After preparation, the pH values of the formulations were determined without dilution by inserting the probe directly into the liquid formulation at room temperature. A calibrated potentiometer B-474 (Micronal, Brazil) was equipped with an Ag electrode/AgCl (Analion V620). 

#### 4.3.2. Electrophoretic Mobility and Zeta Potential

Zeta potential values were determined by measuring the electrophoretic mobility using a Zetasizer Nano ZS (Malvern Instruments Ltd., Malvern, UK). The samples were diluted using 10 mmol L^−1^ NaCl aqueous solution (1:500, *v/v*), and the analysis was carried out at 25 °C.

#### 4.3.3. Laser Diffraction

The granulometric profiles of the DIC-LNC and LNC formulations were obtained using laser diffraction (Malvern Mastersizer 2000, Malvern Instruments, Malvern, UK) in the range of 40 nm to 2 mm. Each sample was added directly to a wet unit containing distilled water (approximately 150 mL) at room temperature. Formulations were analyzed using an obscuration between 2% and 8%. The volume-weighted mean diameter (D[4,3]) and the polydispersity of the distribution (span, based on the diameters at 10%, 50%, and 90% of the cumulative size distribution) were determined.

#### 4.3.4. Dynamic Light Scattering

Formulations were analyzed, at 25 °C, by dynamic light scattering (DLS) using ZetaSizer Nano ZS (Malvern Instruments Ltd., Malvern, UK) to determine the distribution curve of diameters in the range from 1 nm to 5 μm, the mean hydrodynamic diameter (Dh), using the method of Cumulants, and the polydispersity index, using the relative variance in the particle size distribution. The formulations were diluted 500 times in ultrapure water (MilliQ^®^), and the intensity of the scattered light was analyzed at an angle of 173°, providing correlation curves, which were fitted using a monoexponential model.

#### 4.3.5. Nanoparticle Tracking Analysis

Nanoparticle Tracking Analysis (NTA) was performed using a NanoSight instrument (LM10, NanoSight Ltd., Amesbury, UK) after the formulations were diluted (5000×) in pre-filtered ultrapure water (MilliQ^®^). Based on the Brownian motion of the lipid-core nanocapsules, the hydrodynamic diameter (Dh), median diameter (D50), diameter at the 90th percentile (D90) under the cumulative size distribution, and particle number density (PND) were obtained in real time using a CCD camera. The video clips were captured over 60 s at 21.6 ± 0.5 °C and 0.96 ± 0.02 cP.

#### 4.3.6. Drug Content and Encapsulation Efficiency

The DIC content of DIC-LNC was determined by a direct quantification using high-performance liquid chromatography (HPLC, Perkin Elmer S-200 with an S-200 injector) dotted of a UV-VIS detector and mobile phase consisted of acetonitrile: water (65:35 *v/v*) adjusted to an apparent pH of 5.0 ± 0.5 with 10% (*v/v*) acetic acid, with a flow rate of 1.0 mL min^−1^ and injection volume of 20 μL. The sample was diluted in acetonitrile in a volumetric flask and filtered with 0.45 μm membranes (Millipore, Burlington, MA, USA). The methodology presented proper linear regression, with *r* > 0.999 in the interval used, 1–50 μg mL^−1^, and demonstrated specificity, accuracy (99 ± 1%), repeatability, and precision (relative standard deviation <5%).

The encapsulation efficiency (EE%) was established by indirect quantification using ultrafiltration–centrifugation by adding 300 μL of the formulation, without previous dilution, to a Microcon^®^ centrifugal filter device (10 KDa, Millipore^®^ USA), after which the unit was centrifuged at 1844× *g* (RCF) for five min using a centrifuge (Sigma^®^ 1-14, Osterode, Germany). The ultrafiltrate was analyzed by HPLC as described above. The EE% was calculated as the difference between the total drug content (total concentration of the drug in the formulation) and the drug concentration in the ultrafiltrate (concentration of the dissolved drug in the continuous phase), divided by the total content, and multiplied by 100. All analyses were performed in triplicate.

### 4.4. Animals

Twenty 8-week-old Lewis rats weighing 250–350 g were maintained in a biotherium at the Federal University of Amazonas (UFAM) and used in the experiment. The animals had access to water and food and were maintained in light-controlled (12 h light–dark cycle) and temperature-controlled (22 °C) environments. They were allocated by simple randomization into four groups: four animals were maintained in each cage, and one animal was kept alone as a negative control. All experiments were approved by the Institutional Committee for Ethics in Animal Experiments under reference number 010/2010 (CEEA) UFAM.

### 4.5. Induction of Arthritis by Freund’s Complete Adjuvant and Experimental Design

The experimental design is shown in the Graphical Abstract. Arthritis was induced through an intradermal injection on the tail of the rats with 0.1 mL of complete Freund’s adjuvant (BD Difco^TM^, Franklin Lakes, NJ, USA) into the tail of the rats after inhalation of isoflurane anesthesia on the first day of the experiment. After evidence of arthritis, the animals were allocated into four groups using simple randomization: Group 1, control (arthritic rats without treatment); Group 2, arthritis with empty LNC; Group 3, arthritis with a free solution of diclofenac; and Group 4, arthritis with the nanoformulation DIC-LNCs. On day 0 of the experiment, each treatment was initiated with an intraperitoneal injection of DIC, DIC-LNC, and empty LNC, respectively, for each group. The DIC and DIC-LNC doses were 3 mg/kg/day. On day 28 of the experiment, the animals were euthanized by isoflurane inhalation, and blood was obtained by heart puncture.

### 4.6. Randomization, Blinding, and Allocation Concealment

A list of random numbers was used to allocate the animals to a treatment group, and the animals were raffled by simple randomization (randomization.com.br, accessed on 1 May 2023). Allocation concealment was achieved using a codified random list produced by personnel not involved in the experimental design or conduct. All animal treatment assessments, biochemical markers, and stereological data analyses were performed under blinded conditions.

### 4.7. Edema Volume and Arthritis Score of the Hind Paw

Paw volumes were measured on days 0, 7, 14, 21, and 28 of the experiment using a digital plethysmometer (Insight^®^, Ribeirão Preto, Brazil). A blinded evaluator assessed paw volume. The arthritis score was evaluated on the 28th day of the experiment. Rat paws were scored using an arthritis score as previously described [48].

### 4.8. Quantification of Cytokines and Biochemical Markers

With a CBA Flex Sets^®^ (cytometry bead array), the serum levels of TNF-α, IL-1α, and CRP were measured, following the manufacturer’s instructions. A FACSCalibur flow cytometer (BD Biosciences) was used to analyze the samples. The quantity and mean fluorescence of the cytokines were calculated using BD FCAP Array™ Software (v 1.0.1; BD Biosciences). A Roche Hitachi Chemistry Analyzer and an immunoturbidimetric assay (catalog number: 4956842190) were used to calculate the serum levels of CRP, following the manufacturer’s instructions. Liver and renal toxicities were evaluated by measuring glutamic oxaloacetic transaminase (TGO), glutamic pyruvic transaminase (TGP), gamma-glutamyl transpeptidase (GGT, creatinine, and blood urea nitrogen (BUN).

### 4.9. Stereological Analysis

After euthanasia, the left and right hind paws were removed and preserved in buffered formaldehyde 10% for further stereological analyses. Each paw was codified according to group allocation, and all stereological analyses were performed using the allocation concealment strategy. The rat paws were washed with distilled water to remove any residual formaldehyde. The second digit of the paws was removed because the articulation of interest was the metatarsophalangeal (MTP) of the second digit. The hair and skin of the digits were peeled to better absorb the decalcifying substance. All the digits were stored in 10% formic acid for 48 h until complete decalcification.

After this, they were washed with distilled water and dehydrated in an alcohol series (70% and 96%), following resin embedding (Technovit 7100; Külzer-Heraeus, Hanau, Germany). Each block containing a finger was stored at 50 °C for 24 h.

**Figure 8:**
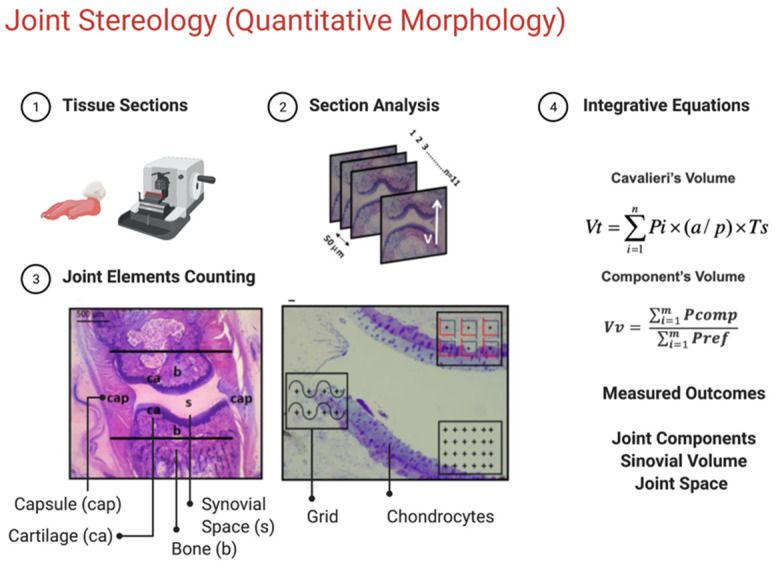
Methods used for stereological analysis. (1) Sectioning level of the 2nd digit of the paw. (2) Block of resin containing an MTP joint (longitudinal lines indicate the location of the knife during the microtomy. Generation of 50 μm equidistant serial sections. Any section passing through the longitudinal plane of the finger will be a vertical section. (3) Profile of a section used to determine Cavalieri volume. In this magnification, the structures marked in the image are fully visible. The lines delimit the area investigated in this study limited by the border with the spongy bone. Detail of the joint region used to estimate the volume density (point-counting system), surface area (counting system with points and curves), and several chondrocyte profiles (counting system with frames and a central point). cap, capsule; b, bone; ca, cartilage; s, synovial space. (4) Integrative equations for quantitative stereology.

Subsequently, each block was positioned on an angle clock and randomly rotated to create different sectioning angles according to the principle of vertical sectioning [49]. Figure 8 shows the procedure used to obtain the vertical sections of the MTP joint. Ten to eleven sections (5 µm thick) were taken with a constant distance (T) of 50 µm for each block using a microtome (Leica RM 2145, Weltzlar, Germany). The sections were stained with toluidine blue 0.5% (toluidine blue, 0.12 g; Na^+^ borate, 0.5 g; distilled H_2_O, 100 mL) and basic fuchsin (basic fuchsin, 0.5 g and distilled H_2_O, 100 mL).

### 4.10. Determination of Cavalieri’s Volume

The volume of the MTP joint was determined according to Cavalieri’s principle [50]. This principle is used when the precise calculation of any volume is required. All serial sections of each block were digitized using a stereomicroscope (Leica EZ4D Digital System, Germany). Through the program Imod 4.7/stereology [51], a counting system of points was overlapped on each section. The procedure consisted of counting all points hitting the MTP joint (defined by the limits of the capsule). The volume was estimated using the following formula:

$$V\;\left({\mathrm{mm}}^{3}\right)=\overset{m}{\underset{i=1}{\sum }}\;\mathrm{Pi}\;\times T\;\times a/p$$

where V was the absolute volume of the MTP joint, ∑Pi was the sum of points hitting MTP, a/p was the area occupied by each central point (23,470 µm^2^), and T (50 µm) was the distance between each section [50].

### 4.11. Determination of Relative Volume

The percentage of each component within the MTP was determined using the Delesse principle [52]. Microscopic fields of view within the joints were systematically, uniformly, and randomly sampled (200× magnification). Following the same procedure, a point-counting system was superimposed onto the images. The percentage of the volume occupied by each component related to the reference space (joint) was calculated as follows:

$$\mathrm{Vv}\;\left(\mathrm{component},\;\mathrm{reference}\;\mathrm{space}\right)=\frac{\sum_{i=1}^{m}\;\mathrm{Pcomp}}{\sum_{i=1}^{m}\;\mathrm{Pref}}$$

where Vv was the volume density of the articular component, ∑Pcomp was the sum of points hitting each component (capsule, synovial space, and synovial membrane), and ∑Pref was the sum of points hitting the reference space (articular region) [52]. The percentage values obtained for each component were transformed into absolute values when multiplied by Cavalieri’s joint volume, as shown in the following formula:

$$\mathrm{Absolut}\;\mathrm{Volume}\left({\mathrm{mm}}^{3}\right)=\mathrm{Vv}\;\times \;\mathrm{Cavalieri}\;\mathrm{Volume}$$

### 4.12. Determination of Surface Area

The same images from the preview calculation of the Delesse volume were used to obtain the articular cartilage and synovial membrane surface areas. Again, a counting system with long cycloid curves and points was superimposed on the images. Each time a curve intercepted the edge of the cartilage in contact with the synovial space or synovial membrane, it was counted. The same occurred at points that touched these structures. The density of the surface was calculated as:

$$\mathrm{Sv}\;\left({\mathrm{mm}}^{-1}\right)=\frac{2\sum_{i=1}^{m}\;I}{\sum_{i=1}^{m}\;\mathrm{Pi}\;\times \frac{l}{p}}$$

where Sv stands for surface density, ∑I is the sum of intersections, ∑P is the sum of points, and l/p is the length of the test curve per grid point. The relative values obtained for each component were transformed into absolute values when multiplied by the Cavalieri volume of the joint as follows:

$$\mathrm{Surface}\;\mathrm{area}\;\left({\mathrm{mm}}^{-1}\right)=\mathrm{Sv}\;\times \;\mathrm{Cavalieri}\;\mathrm{Volume}$$

### 4.13. Counting the Number of Cellular Profiles

To determine the number of chondrocyte profiles present in the articular cartilage, we used a 2-D quantification technique. The counting system comprises six frames (2700.28 µm^2^), each containing a reference point. The counting frames have a solid forbidden line and a dashed acceptance line. Only the nuclei of cells that appeared inside the counting frame and did not touch the exclusive line were counted. The number of cells was expressed as profiles/mm^2^ and calculated using the following formula:

$$\mathrm{Nv}=\frac{\sum_{i=1}^{m}\;Q}{\mathrm{Nf}\;\times \;\mathrm{Af}}$$

where ∑Q was the cellular profile number of chondrocytes, ∑N frames were the sum of all the analyzed molding frames, Af was the area of the molding frame.

### 4.14. Statistical Analysis

Data are expressed as means and standard deviation. The results are expressed as the mean and standard deviation, and a 95% confidence interval was used. One-way or two-way analysis of variance (ANOVA) was used to compare means, and Sidak’s test for multiple comparisons or a linear test for trend was used for post hoc analysis. The Mann–Whitney U test was used when Bartlett’s analysis did not observe normality. Linear regression and curve fitting with nonlinear polynomial regression were applied to study the effects of different MTX preparations on cytokine levels in cultured blood and synovial cells. A significance level of α = 0.05 was adopted, and all *p*-values were two-tailed. For convenience, at the figures the number of asterisks over the bars are the number of digits with significance after the decimal point. The level of significance used was 5%. For the stereological analysis, the variance was expressed as the coefficient of error (CE) for each parameter evaluated (V, Vv, and Sv). The accuracy of the Cavalieri volume estimates was determined according to [53],

$$\mathrm{CE}={\left[0.0724\times \frac{B}{\sqrt{A}}\times \frac{\sqrt{n}}{{\left(\sum_{i=1}^{m}\;\mathrm{Pi}\right)}^{\frac{3}{2}}}\right]}^{\frac{1}{2}}$$

where CE indicates the coefficient of error for Cavalieri’s volume determination, $\frac{B}{\sqrt{A}}\;$ Indicates the variance in the count of the sections (shape coefficient), which depends on the morphological complexity of the structure, n represents the number of sections evaluated, and is the number of points counted on the sections.

## 5. Conclusions

Our results demonstrate the anti-arthritic activity of DIC-LNCs. Using stereological analysis, we calculated the articular volume of the MTP and its components, the surface areas of the cartilage and synovium, and the 2-D cellular count profile of chondrocytes. We demonstrated that lipid-core nanocapsules reduced (1) edema and joint synovitis (2) inflammatory markers and preserved the articular cartilage, chondrocytes, and synovial space (4) without any obvious toxicity sign to the liver or kidneys. Thus, DIC-LNCs are innovative and promising nanoformulations for treating RA and other inflammatory joint diseases. However, further analysis is required to determine the effects of nanocapsules on humans.

## Figures and Tables

**Table 1:** Characterization of the formulations by potentiometry, laser diffraction, dynamic light scattering, and nanoparticle tracking analysis.

	LNC	DIC-LNC
Potentiometry		
pH	5.43 ± 0.24	5.39 ± 0.16
Laser diffraction		
D[4,3] (nm)	153 ± 10	204 ± 46
Span	1.4 ± 0.2	1.7 ± 0.1
Dynamic Light Scattering		
Dh (nm)	170 ± 13	166 ± 13
PDI	0.06 ± 0.02	0.08 ± 0.02
Electrophoretic mobility		
Zeta Potential (mV)	−13 ± 6	−11 ± 2
Nanoparticle tracking analysis		
Dh (nm)	182 ± 9	196 ± 14
D50 (nm)	173 ± 13	186 ± 2
D90 (nm)	257 ± 10	309 ± 9
PND (× 10^12^ particles mL^−1^)	4.98 ± 0.25	4.76 ± 0.78

Note. Data are expressed as mean ± standard deviation. Abbreviations: LNC, blank lipid-core nanocapsules; DIC-LNC, acid diclofenac-loaded lipid-core nanocapsules; D[4,3], volume-weighted diameter average; Dh, hydrodynamic diameter; PDI, polydispersity index; D50, median diameter; D90, the diameter at the 90th percentile; PND, particle number density.

**Table 2:** MTP joint volume and density of its different components.

	Volume (mm^3^)					
Groups	Joint	Cartilage	Bone	Capsule	Synovial Space	Synovial Membrane
Arthritis	3.02 ± 0.08	0.50 ± 0.10	1.20 ± 0.05	0.97 ± 0.14	0.16 ± 0.01	0.08 ± 0.02
LNC	2.80 ± 0.32	0.36 ± 0.08	1.08 ± 0.16	0.89 ± 0.15	0.17 ± 0.01	0.04 ± 0.01
Diclofenac	2.46 ± 0.12	0.34 ± 0.03	1.02 ± 0.12	0.67 ± 0.02	0.19 ± 0.02	0.03 ± 0.00
Diclofenac-LNC	2.10 ± 0.10	0.21 ± 0.01	0.75 ± 0.05	0.58 ± 0.02	0.26 ± 0.01	0.02 ± 0.00
No Arthritis	1.95 ± 0.05	0.23 ± 0.03	0.61 ± 0.06	0.52 ± 0.03	0.41 ± 0.03	0.01 ± 0.05

Note. Data are expressed as mean ± standard deviation. ANOVA and Sidak’s test multiple comparison tests (*p* < 0.001). Abbreviations: LNC, blank lipid-core nanocapsules and Sodium Diclofenac-LNC, Diclofenac loaded lipid-core nanocapsules.

**Table 3:** Surface area and cellular count of MTP.

	Surface Area (mm^2^)		Cellular Count (Cell/mm^2^)		
Groups	Cartilage	Synovial Membrane	Chondrocytes	Isogenous Groups	Total Chondrocytes
Arthritis	38.25 ± 4.32	13.26 ± 2.78	1416 ± 126.3	80.55 ± 8.77	1517 ± 54.30
LNC	29.09 ± 3.75	9.98 ± 1.49	1543 ± 49.92	111.5 ± 4.51	1701 ± 91.96
Diclofenac	27.87 ± 3.31	9.92 ± 0.46	1627 ± 95.61	161.1 ± 11.84	1849 ± 16.73
Diclofenac-LNC	20.45 ± 1.58	8.01 ± 0.19	1894 ± 47.00	226.8 ± 20.5	2095 ± 52.33
No Arthritis	19.45 ± 0.28	7.43 ± 1.04	2208 ± 217.4	297.9 ± 19.5	2367 ± 225.8

Note: Data are expressed as mean ± standard deviation. Abbreviations: LNC, blank lipid-core nanocapsules and Sodium Diclofenac-LNC, Diclofenac loaded lipid-core nanocapsules

## Data Availability

All data and materials in this manuscript are available in the final version of the manuscript.
